# Inter-assay diagnostic accuracy of cerebrospinal fluid kappa free light chains for the diagnosis of multiple sclerosis

**DOI:** 10.3389/fimmu.2024.1385231

**Published:** 2024-04-30

**Authors:** Cathérine Dekeyser, Pieter De Kesel, Melissa Cambron, Ludo Vanopdenbosch, Liesbeth Van Hijfte, Martine Vercammen, Guy Laureys

**Affiliations:** ^1^Department of Neurology, Ghent University Hospital, Ghent, Belgium; ^2^Department of Laboratory Medicine, Ghent University Hospital, Ghent, Belgium; ^3^Department of Neurology, AZ Sint-Jan Brugge, Bruges, Belgium; ^4^Faculty of Medicine and Health Sciences, Ghent University, Ghent, Belgium; ^5^Department of Laboratory Medicine, Algemeen Ziekenhuis (AZ) Sint-Jan Brugge, Bruges, Belgium; ^6^Basic Biomedical Sciences, Vrije Universiteit Brussel, Brussels, Belgium

**Keywords:** multiple sclerosis, kappa free light chains, κFLC index, κIgG index, CSF κFLC/IgG ratio, Freelite^®^-Optilite versus N Latex^®^-BNII

## Abstract

**Background:**

Cerebrospinal fluid (CSF) kappa free light chain (κFLC) measures gained increasing interest as diagnostic markers in multiple sclerosis (MS). However, the lack of studies comparing assay-dependent diagnostic cutoff values hinders their use in clinical practice. Additionally, the optimal κFLC parameter for identifying MS remains a subject of ongoing debate.

**Objectives:**

The aim of this study was to compare same-sample diagnostic accuracies of the κFLC index, κIgG index, CSF κFLC/IgG ratio, and isolated CSF κFLC (iCSF-κFLC) between two reference centers using different methods.

**Methods:**

Paired serum and CSF samples were analyzed for κFLC and albumin concentrations by Freelite^®^-Optilite (Sint-Jan Bruges hospital) and N Latex^®^-BNII (Ghent University hospital). Diagnostic performance to differentiate MS from controls was assessed using ROC curve analysis.

**Results:**

A total of 263 participants were included (MS, *n* = 80). Optimal diagnostic cutoff values for the κFLC index (Freelite^®^-Optilite: 7.7; N Latex^®^-BNII: 4.71), κIgG index (Freelite^®^-Optilite: 14.15, N Latex^®^-BNII: 12.19), and CSF κFLC/IgG ratio (Freelite^®^-Optilite: 2.27; N Latex^®^-BNII: 1.44) differed between the two methods. Sensitivities related to optimal cutoff values were 89.9% (Freelite^®^-Optilite) versus 94.6% (N Latex^®^-BNII) for the κFLC index, 91% (Freelite^®^-Optilite) versus 92.2% (N Latex^®^-BNII) for the κIgG index, and 81.3% (Freelite^®^-Optilite) versus 91.4% (N Latex^®^-BNII) for the CSF κFLC/IgG ratio. However, for iCSF-κFLC, optimal diagnostic cutoff values (0.36 mg/L) and related specificities (81.8%) were identical with a related diagnostic sensitivity of 89.9% for Freelite^®^-Optilite and 90.5% for N Latex^®^-BNII. The diagnostic performance of the κFLC index [area under the curve (AUC) Freelite^®^-Optilite: 0.924; N Latex^®^-BNII: 0.962] and κIgG index (AUC Freelite^®^-Optilite: 0.929; N Latex^®^-BNII: 0.961) was superior compared to CSF oligoclonal bands (AUC: 0.898, sensitivity: 83.8%, specificity: 95.9%).

**Conclusions:**

The κFLC index and the κIgG index seem to be excellent markers for identifying MS, irrespective of the method used for κFLC quantification. Based on the AUC, they appear to be the measures of choice. For all measures, optimal cutoff values differed between methods except for iCSF-κFLC. iCSF-κFLC might therefore serve as a method-independent, more cost-efficient, initial screening measure for MS. These findings are particularly relevant for clinical practice given the potential future implementation of intrathecal κFLC synthesis in MS diagnostic criteria and for future multicentre studies pooling data on κFLC measures.

## 1 Introduction

Multiple sclerosis (MS) is an inflammatory and degenerative disorder of the central nervous system (CNS), characterized by focal inflammatory lesions and diffuse neurodegeneration (1). The diagnosis of MS requires the combination of clinical signs, symptoms, and paraclinical findings obtained by magnetic resonance imaging (MRI) and/or cerebrospinal fluid (CSF) analysis. In the appropriate clinical setting, the diagnostic certainty can be increased by the demonstration of intrathecal immunoglobulin G (IgG) synthesis. The presence of CSF oligoclonal bands (OCBs) is considered the gold standard in this regard and currently substitutes for dissemination in time according to the 2017 McDonald criteria (2). Although the value of CSF OCBs detection is indisputable in MS, it suffers from well-known disadvantages as it is a time-consuming, subjective, and qualitative method with a challenging interpretation. Plasma cells and plasma blasts produce intact immunoglobulins consisting of light and heavy chains bound together via disulfide bonds. Besides intact immunoglobulins, they also produce light chains in 10%–40% excess over heavy chains and secrete them as free forms in the blood circulation. In case of chronic inflammatory neurological disease of the CNS such as MS, kappa free light chains (κFLC) are produced intrathecally (3, 4).

Although the presence of κFLC in the CSF of persons with MS (PwMS) was already assumed in 1974 (5), only recently did they gain increasing interest as alternative markers reflecting intrathecal IgG synthesis. Various CSF κFLC parameters have been proposed; however, the ideal κFLC parameter remains a matter of debate. The majority of the published studies focused on the κFLC index (6, 7) and consistently demonstrated its high diagnostic sensitivity and specificity of approximately 90%, similar to CSF-restricted OCBs. Other authors investigated the diagnostic value of the κIgG index (8), CSF κFLC/IgG ratio (9), or isolated CSF κFLC (10). Evidence on the comparison of different κFLC parameters to distinguish MS from controls remains scarce and is currently limited to a few studies in which conflicting results on the superiority of one κFLC measure over the other were reported (10–20). In addition, there is a lack of consensus on diagnostic cutoff values to differentiate MS from other neurological conditions as published diagnostic κFLC index cutoff values, for instance, range from 2.4 to 20 (7).

κFLC are usually measured by their automated turbidimetric or nephelometric quantification using one of the commercially available κFLC immunoassays (7), of which the polyclonal Freelite^®^ (The Binding Site) and monoclonal N Latex^®^ (Siemens Healthineers) assay are the most widely used. In patients with different plasma cell dyscrasias, comparisons between Freelite^®^ and N Latex^®^ κFLC immunoassays revealed substantially equivalent clinical performance (21–28), although differences in absolute values were consistently demonstrated (24, 25, 27–31). Similar evidence on inter-assay variability is lacking for κFLC parameters reflecting intrathecal IgG synthesis. Since the use of different commercial assays to detect κFLC might hamper the interchangeability of the results, there is a need for multicenter studies assessing the between-method diagnostic accuracy and between-method optimal cutoff values of various κFLC parameters.

## 2 Materials and methods

### 2.1 Patients and samples

This is a multicenter study with Ghent University Hospital (GUH) and Sint-Jan Bruges Hospital (SJB) as participating centers. All patients in whom a lumbar puncture (LP) was performed during routine clinical practice were eligible to participate. Consecutive paired serum and CSF samples with a residual volume of at least 400 µL stored at −80°C in the biobanks of either GUH or SJB from February 2018 until the 14 September 2023 were used for analysis. In total, 263 paired serum and CSF samples were included. Participants were divided into four groups according to their diagnosis: (1) MS (*n* = 80): in order to be assigned to the MS group, the revised 2017 McDonald criteria needed to be fulfilled (2); (2) other inflammatory or infectious neurological diseases of the central and peripheral nervous system (OIND, *n* = 51); (3) non-inflammatory neurological diseases (NIND; *n* = 102); and (4) symptomatic controls (no evidence of organic central or peripheral nervous system disease, SC, *n* = 30). This categorization is in line with the one proposed by Teunissen et al. (32).

The diagnoses in the OIND group comprised infectious meningoencephalitis (*n* = 7), aseptic meningitis (*n* = 2), auto-immune or paraneoplastic encephalitis (*n* = 13), acute disseminated encephalomyelitis (*n* = 1), myelitis (*n* = 2), cerebral abscess (*n* = 2), Susac syndrome (*n* = 3), neuroborreliosis (*n* = 1), plexitis (*n* = 1), immune-mediated polyneuropathies (*n* = 5), cryptogenic optic neuritis (*n* = 3), neurosarcoidosis (*n* = 2), Tacrolimus-induced demyelination (*n* = 1), inflammatory or infectious (poly)neuritis cranialis (*n* = 5), CNS vasculitis (*n* = 2), and myelin oligodendrocyte glycoprotein-associated disorder (*n* = 1). In NIND, the diagnoses comprised stroke (*n* = 17), reversible vasoconstriction syndrome (*n* = 1), vascular white matter lesions (*n* = 8), venous sinus thrombosis (*n* = 1), amyotrophic lateral sclerosis (*n* = 3), cerebellar neurodegeneration (*n* = 2), non-infectious or non-inflammatory myelopathy (*n* = 5), spinocerebellar ataxia (*n* = 1), transient global amnesia (*n* = 1), metabolic or hereditary leukodystrophies (*n* = 2), epilepsy of non-inflammatory cause (*n* = 12), disturbed consciousness due to carbon dioxide narcosis (*n* = 1), non-inflammatory polyneuropathy (*n* = 2), paramyotonia congenita (*n* = 1), idiopathic intracranial hypertension (*n* = 3), normotensive hydrocephalus (*n* = 3), dementia disorders and Parkinson syndromes (*n* = 27), narcolepsy type 1 (*n* = 2), narcolepsy type 2 (*n* = 2), Marchiafava–Bignami disease (*n* = 1), diffuse axonal injury (*n* = 1), migraine (*n* = 5), and non-infectious, non-inflammatory bilateral optic neuropathy (*n* = 1). Finally, in the SC group, the majority of the LPs were performed to exclude meningoencephalitis (*n* = 18) or other neurological disorders such as narcolepsy (*n* = 1) or Vogt–Koyanagi–Harada syndrome (*n* = 1) or were performed in the context of polymorphic complaints (*n* = 2). In all these patients, the diagnostic workup could not provide sufficient evidence for an underlying neurological disorder. Alternative disorders in the SC group comprised functional disorders (*n* = 6) and hyperventilation (*n* = 2).

### 2.2 Ethics approval

This study was approved by the local Ethics Committees of the participating centers (Ref. 2022-0150) and was performed in accordance with the ethical standards laid down in the 1964 Declaration of Helsinki and its later amendments. All patients were informed about the anonymized use of the surplus of the fluids for research purposes.

### 2.3 Methods

CSF κFLC, serum κFLC, CSF albumin, and serum albumin levels were quantified on each paired serum/CSF sample using different combinations of commercially available κFLC assays and analyzers available in the two participating centers:

1. Immunoturbidimetry with the Freelite^®^ reagent on Optilite (The Binding Site, Birmingham, UK, SJB)
2. Immunonephelometry with the N Latex^®^ reagent on BNII(Siemens Healthcare Diagnostic Products, GUH)

CSF was drawn by LP and venous blood was drawn by venipuncture under standard conditions. Immediately after all routine tests had been performed, paired CSF and serum samples were frozen to −80°C. Samples were thawed only once before κFLC and albumin quantitative analysis. Analyses in the two centers were performed on the same day.

In the majority of participants, oligoclonal band status (presence or absence) (*n* = 252) and CSF (*n* = 247) and serum (*n* = 239) IgG levels were determined as per clinical routine at the time of sample collection. In both centers, OCBs status was determined by isoelectric focusing (Hydragel CSF Isoelectric focusing kit on Hydrasys, Sebia). OCBs were considered positive if two or more CSF-restricted OCBs were present. Serum and CSF IgG levels were determined by nephelometry using the N Antiserum to Human IgG kit on BN II in GUH. In SJB, serum IgG levels were determined by the Atellica CH IgG kit, whereas CSF IgG levels were determined by the CSF IgG DiAgam kit on Atellica CH. Information on OCBs status and serum and CSF IgG levels at the time of sample collection was collected from the electronic patient file.

#### 2.3.1 Immunoturbidimetry

In SJB, turbidimetric analysis of κFLC in serum and CSF as well as albumin in serum and CSF was performed on the Optilite platform (The Binding Site Ltd., Birmingham, UK) using The Binding Site reagent, according to the manufacturer’s instructions. The Freelite^®^ Mx Kappa Free Kit for both CSF and serum consists of polyclonal sheep antibodies coated onto polystyrene latex, to enhance the reaction and allowing amplification of the signal. Freelite^®^ reacts only with exposed free light chain epitopes, which are hidden when the light chain is bound to the heavy chain. The Freelite^®^ κFLC measuring range in both serum and CSF is 0.33–127,000 mg/L, with an assay detection limit of 0.27 mg/L in CSF. As for albumin, the Low-Level Albumin kit for CSF and serum (measuring range 11–66,500 mg/L) was used.

#### 2.3.2 Immunonephelometry

Nephelometric analysis was performed with the BN II System in GUH (BNII Siemens Healthineers Diagnostic Products GmbH, Marburg, Germany), according to the manufacturer’s instructions. Siemens reagent for κFLC is the N Latex^®^ FLC kappa assay in both CSF (linear range, 0.034–110 mg/L) and serum (linear range, 0.195–110 mg/L) and consists of a mixture of mouse monoclonal antibodies covalently coupled to polystyrene particles. The reagent is based on the principle of particle-enhanced immunonephelometry. As for albumin, the N Albumin antiserum anti-human albumin kit in CSF (linear range, 17.0–110,000 mg/L) and serum (linear range, 355-110,000 mg/L) was used.

All assays were verified in accordance with local quality requirements of the ISO15189-accredited medical laboratory. GUH participates in an external quality assessment scheme of the Reference Institute of Bioanalytics (Bonn, Germany) for all analytes, whereas SJB participates in the UK NEQAS (Sheffield, UK) external quality assessment.

#### 2.3.3 κFLC index, κIgG index, and CSF κFLC/IgG ratio

The κFLC index, κIgG index, and CSF κFLC/IgG ratio were calculated using the following formulas: [CSF κFLC (mg/L)/serum κFLC (mg/L)]/[CSF albumin (mg/L)/serum albumin (mg/L)], [CSF κFLC (mg/L)/serum κFLC (mg/L)]/[CSF IgG (mg/L)/serum IgG (mg/L)], and [CSF κFLC (mg/L)/CSF IgG (mg/L)] × 100.

### 2.4 Statistical analysis

Statistical analysis was performed to evaluate differences, correlation, and grade of concordance between the results obtained with Freelite^®^-Optilite versus N Latex^®^-BNII. The diagnostic accuracy to differentiate MS from controls was calculated as the area under the receiver operating characteristics (ROC) curve (AUC). Optimal diagnostic cutoff values of different κFLC parameters were determined using maximization of the Youden index. Diagnostic cutoff values are reported with their respective sensitivities, specificities, and likelihood ratios (sensitivity/(1 − specificity)) (LR). Differences between values obtained with Freelite^®^-Optilite versus N Latex^®^-BNII were sought using the Wilcoxon signed rank test for all analytes. Values were expressed as median and interquartile ranges (IQR). Quantitative method comparison was performed by Passing–Bablok regression and Bland–Altman analysis. Absolute individual agreement between values obtained with Freelite^®^-Optilite versus N Latex^®^-BNII were calculated using a two-way mixed-effects intraclass correlation coefficient (ICC). ICC values <0.5 were considered indicative for poor reliability, 0.5 < ICC < 0.75 for moderate reliability, 0.75 < ICC < 0.9 for good reliability, and ICC > 0.9 for excellent reliability. Associations between patient-related factors such as age, sex, corticosteroid administration, and sample storage duration on κFLC measures were assessed using Spearman rank correlation and multivariable linear regression analysis where appropriate. All analyses were performed using SPSS statistical software version 29.0 except for Passing–Bablok regression and Bland–Altman analysis, which was performed using MedCalc. *p*-values less than 0.05 were considered statistically significant.

## 3 Results

### 3.1 Comparison of the diagnostic accuracy of isolated CSF κFLC, the κFLC index, κIgG index, and CSF κFLC/IgG ratio

**Figure 1:**
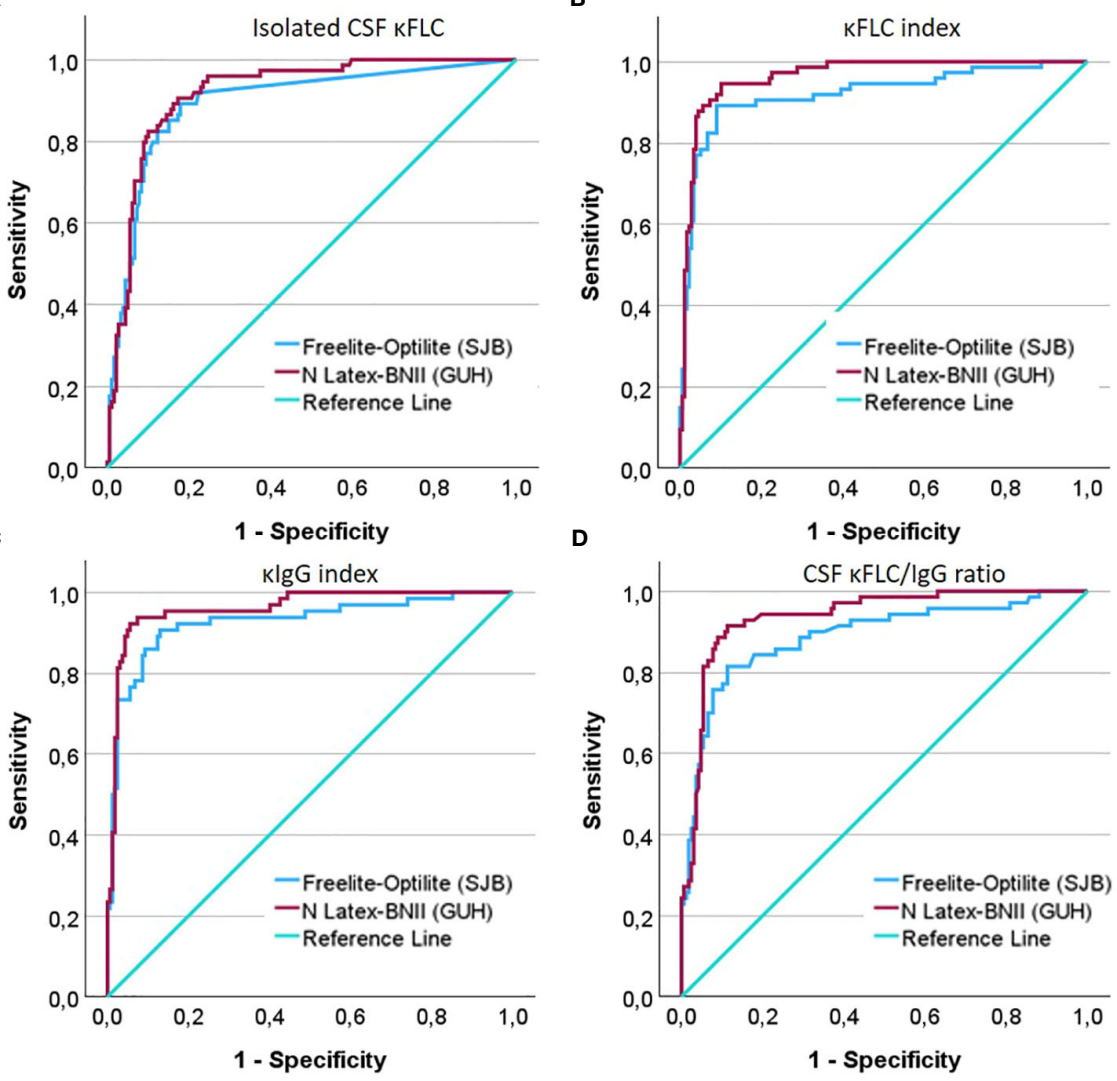
ROC curves demonstrating the diagnostic accuracy of **(A)** isolated CSF κFLC (mg/L), **(B)** the κFLC index **(C)**, the κIgG index, and **(D)** the CSF κFLC/IgG ratio to differentiate MS from controls.

The comparison between the diagnostic accuracies and optimal cutoff values of the κFLC parameters obtained with Freelite^®^-Optilite versus N Latex^®^-BNII is presented in **Table 1** and **Figure 1**. CSF κFLC concentrations were below the detection limit in 146 (MS = 6, OIND = 34, NIND = 78, and SC = 28) and 4 participants (OIND = 2, NIND = 1, and SC = 1) for Freelite^®^-Optilite (detection limit, 0.27 mg/L) and N Latex^®^-BNII (detection limit, 0.035 mg/L), respectively. Undetectable CSF κFLC levels were replaced by the detection limit. The sensitivities, specificities, and LR corresponding to a κFLC index cutoff value of 6.1 are also displayed in **Table 1**, as 6.1 was determined as the discriminatory diagnostic cutoff value in a recent meta-analysis including 32 studies (33). In addition, the diagnostic cutoff values corresponding to the LR closest to 5 were determined for Freelite^®^-Optilite and N Latex^®^-BNII, reflecting the cutoff value corresponding to a five times higher probability of MS versus controls (**Supplementary Table 1**).

**Table 1:** Diagnostic accuracy of the κFLC parameters using Freelite^®^-Optilite and N Latex^®^-BNII: Optimal cutoff values as determined by maximization of the Youden index.

Freelite-Optilite (Bruges)						
MS vs. no Ms						
	Cutoff from the ROC	Sensitivity	Specificity	LR	Youden index	AUC
CSF κFLC (mg/L)	0.36	89.9	81.8	4.94	0.716	0.897 (0.853–0.942)
κFLC index (optimal)	7.70	89.9	91.1	10.10	0.809	0.924 (0.884–0.965)
κFLC index	6.1	89.9	83.2	5.35	0.731	0.924 (0.884–0.965)
κIgG index	14.15	91	87.2	7.11	0.782	0.929 (0.887–0.971)
CSF κFLC/IgG ratio	2.27	81.3	88.8	7.26	0.702	0.882 (0.829–0.935)
MS vs. OIND						
CSF κFLC (mg/L)	0.28	92.4	68	2.89	0.604	0.819 (0.738–0.900)
κFLC index (optimal)	7.70	89.9	78	4.09	0.679	0.877 (0.813–0.941)
κIgG index	11.14	92.5	74.4	3.61	0.670	0.888 (0.821–0.954)
CSF κFLC/IgG ratio	1.79	84	71.1	2.90	0.551	0.827 (0.750–0.903)
N Latex-BN II (Ghent)						
MS vs. no Ms						
	Cutoff from the ROC	Sensitivity	Specificity	LR	Youden index	AUC (95% CI)
CSF κFLC (mg/L)	0.36	90.5	81.8	4.97	0.723	0.912 (0.875–0.949)
κFLC index (optimal)	4.71	94.6	89.5	9	0.841	0.962 (0.940–0.984)
κFLC index	6.1	89.2	93.4	13.51	0.826	0.962 (0.940–0.984)
κIgG index	12.19	92.2	94	15.37	0.862	0.961 (0.934–0.988)
CSF κFLC/IgG ratio	1.44	91.4	87.6	7.37	0.791	0.935 (0.902–0.968)
MS versus OIND						
CSF κFLC (mg/L)	0.65	82.4	80	4.12	0.624	0.823 (0.738–0.909)
κFLC index (optimal)	10.64	86.5	86	6.18	0.725	0.910 (0.852–0.968)
κIgG index	12.19	92.2	83.7	5.66	0.759	0.915 (0.856–0.974)
CSF κFLC/IgG ratio	2.64	81.4	82.2	4.57	0.637	0.86 (0.786–0.934)
MS vs. no Ms						
	Cutoff from the ROC	Sensitivity	Specificity	LR	Youden index	AUC
OCBs	Positive	83.8	95.9	20.44	0.797	0.898 (0.848–0.949)
MS versus OIND						
OCBs	Positive	83.8	89.4	7.91	0.731	0.866 (0.796–0.935)

Diagnostic accuracy of the κFLC parameters using Freelite^®^-Optilite and N Latex^®^-BNII. κFLC, kappa free light chains; mg, milligram; L, liter; IgG, immunoglobulin G; MS, multiple sclerosis; OIND, other inflammatory or infectious neurological diseases of the central and peripheral nervous system; ROC, receiver operating characteristics; CI, confidence interval; LR, likelihood ratio; AUC, area under the curve.

The κFLC index, κIgG index, CSF κFLC/IgG ratio, and isolated CSF κFLC determined by N Latex^®^-BNII as well as the κFLC index and κIgG index using Freelite^®^-Optilite demonstrated excellent diagnostic accuracy to differentiate MS from controls (consisting of OIND, NIND, and SC) as illustrated by AUC exceeding 0.90. Good diagnostic power could be demonstrated for isolated CSF κFLC and for the CSF κFLC/IgG ratio with Freelite^®^-Optilite. ROC curve analysis showed that for isolated CSF κFLC, the optimal cutoff value to distinguish MS from controls was 0.36 mg/L with both methods (N Latex^®^-BNII: AUC, 0.912; sensitivity, 90.5%; specificity, 81.8; LR, 4.97; Freelite^®^-Optilite: AUC, 0.897; sensitivity, 89.9%; specificity, 81.8%; LR, 4.94). On the other hand, the κFLC index cutoff value was ≥4.71 (optimized sensitivity, 94.6%; specificity, 89.5%; LR, 9) with N Latex^®^-BNII, whereas this cutoff value was 7.7 with Freelite^®^-Optilite (sensitivity, 89.9%; specificity, 91.1%; LR, 10.10). Similarly, a κIgG index ≥12.19 (sensitivity, 92.2%; specificity, 94%; LR, 15.37) and CSF κFLC/IgG ratio ≥1.44 (sensitivity, 91.4%; specificity, 87.6%; LR, 7.37) showed the best combination of sensitivity and specificity with N Latex^®^-BNII, whereas a κIgG index ≥14.15 (sensitivity, 91%; specificity, 87.2%; LR, 7.11) and a CSF κFLC/IgG ratio ≥2.27 (sensitivity, 81.3%; specificity, 88.8%; LR, 7.26) were optimal cutoff values with Freelite^®^-Optilite.

All κFLC measures further showed good diagnostic power to differentiate MS from OIND with Freelite^®^-Optilite, whereas the diagnostic power ranged from good (isolated CSF κFLC and CSF κFLC/IgG ratio) to excellent (κFLC index and κIgG index) for N Latex^®^-BNII. ROC curve analysis demonstrated that MS could be differentiated from OIND with the highest sensitivity and specificity using a κFLC index ≥10.64, a κIgG index ≥12.19, a CSF κFLC/IgG ratio ≥2.64, and isolated CSF κFLC levels ≥0.65 mg/L using N Latex^®^-BNII, whereas the corresponding values were ≥7.70, ≥11.14, ≥1.79, and ≥0.28 mg/L, respectively, for Freelite^®^-Optilite.

For both methods, the diagnostic power (AUC) of the κFLC index and κIgG index exceeded that of CSF-restricted OCBs. However, compared to the specificities related to the optimal cutoff values obtained by maximization of the Youden index, CSF-restricted OCBs remained the most specific method to confirm a diagnosis of MS (**Table 1**). In **Supplementary Table 2**, the number of participants with MS with both positive CSF OCBs and positive κFLC parameters according to optimal cutoff values as well as discordant results are presented. Of note, of the six participants with MS with undetectable CSF κFLC levels with Freelite^®^-Optilite, one had CSF OCBs.

Overall the diagnostic accuracy seemed slightly better using N Latex^®^-BNII (AUC) compared to Freelite^®^-Optilite as demonstrated by the higher AUC. Because of the interdependency of sensitivity and specificity, the Freelite^®^-Optilite κFLC index was slightly more specific whereas the N Latex^®^-BNII κFLC index was more sensitive to discriminate MS from controls, while the Freelite^®^-Optilite κFLC index was more sensitive and the N Latex^®^-BNII κFLC index was more specific to discriminate MS from OIND at the optimal cutoffs.

### 3.2 Comparison of CSF and serum κFLC and albumin concentrations

CSF and serum κFLC and CSF and serum albumin concentrations measured with the two methods and results of the reliability analysis are presented in **Table 2**. Given the purpose of the study, only measurable values were used for CSF κFLC method comparison. For illustrative purposes, a second analysis was performed where undetectable CSF κFLC levels were replaced by the detection limit (**Supplementary Table 3**).

**Table 2:** Serum and CSF κFLC and albumin levels: Comparison between Freelite^®^-Optilite and N Latex^®^-BNII.

Whole cohort	Freelite-Optilite (Bruges)	N Latex-BN II (Ghent)		Reliability analysis
	Median (IQR)	Median (IQR)	*p*-value	ICC (95% CI)
CSF κFLC (mg/L)	1.73 (0.66–4.09) (*n* = 114)	1.50 (0.63–3.92) (*n* = 108)	0.207	0.982 (0.974–0.988)
Serum κFLC (mg/L)	15.69 (12.09–20.90) (*n* = 261)	13.20 (10.5–17.2) (*n* = 263)	**<0.001**	0.532 (0.439–0.614)
CSF albumin (mg/L)	227.1 (159.25–297.83) (*n* = 260)	234.50 (164.75–316.50) (*n* = 258)	**0.027**	0.989 (0.986–0.992)
Serum albumin (mg/L)	38,917.9 (35,747.05–42,715.20) (*n* = 261)	42,800 (39,500–46,400) (*n* = 263)	**<0.001**	0.80 (−0.24–0.938)
MS	Freelite-Optilite (Bruges)	N Latex-BN II (Ghent)		
CSF κFLC (mg/L)	2.58 (1.00–4.98) (*n* = 73)	2.51 (1.01–4.88) (*n* = 68)	0.355	0.981 (0.969–0.988)
Serum κFLC (mg/L)	13.95 (10.96–18.27) (*n* = 80)	12 (9.42–14.68) (*n* = 80)	<0.001	0.808 (0.068–0.937)
CSF albumin (mg/L)	182.50 (136.7–238.9) (*n* = 79)	235 (185–292) (*n* = 75)	<0.001	0.694 (0.288–0.851)
Serum albumin (mg/L)	41,233.75 (37,765.55–45,553.28) (*n* = 80)	45,700 (41,650–49,075) (*n* = 80)	<0.001	0.700 (−0.55–0.898)
OIND	Freelite-Optilite (Bruges)	N Latex-BN II (Ghent)		
CSF κFLC (mg/L)	2.38 (0.56–4.21) (*n* = 16)	3.05 (0.50–5.33) (*n* = 15)	0.478	0.983 (0.951–0.994)
Serum κFLC (mg/L)	15.06 (12.9–19.96) (*n* = 51)	13.3 (10.40–17.2) (*n* = 51)	<0.001	0.935 (0.843–0.969)
CSF albumin (mg/L)	284.85 (154.23–362.05) (*n* = 50)	311 (150–441) (*n* = 51)	0.013	0.996 (0.991–0.998)
Serum albumin (mg/L)	37,224.4 (32,678.80–40,662.60) (*n* = 51)	40,800 (35,800–46,300) (*n* = 51)	<0.001	0.831 (–0.43–0.956)
NIND	Freelite-Optilite (Bruges)	N Latex-BN II (Ghent)		
CSF κFLC (mg/L)	0.47 (0.34–0.79) (*n* = 23)	0.51 (0.35–0.87) (*n* = 23)	0.465	0.905 (0.791–0.958)
Serum κFLC (mg/L)	17.22 (12.99–23.30) (*n* = 100)	14.9 (11.48–19.93) (*n* = 102)	<0.001	0.499 (0.337–0.632)
CSF albumin (mg/L)	251.2 (175.45–318) (*n* = 101)	227.50 (154.75–325) (*n* = 102)	0.005	0.961 (0.941–0.974)
Serum albumin (mg/L)	38,480.2 (35,491.95–41,431.83) (*n* = 100)	41,900 (38,975–45,025) (*n* = 102)	<0.001	0.795 (−0.16–0.936)
Symptomatic controls	(Freelite-Optilite) Bruges	N Latex-BN II (Ghent)		
CSF κFLC (mg/L)	NR (*n* = 2)	NR (*n* = 2)	NR (*n* = 2)	NR (*n* = 2)
Serum κFLC (mg/L) (*n* = 30)	15.86 (12.16–18.63) (*n* = 30)	13.1 (11.43–16.73) (*n* = 30)	<0.001	0.899 (0.374–0.969)
CSF albumin (mg/L)(*n* = 30)	227.95 (160.73–276.75) (*n* = 30)	219 (146.5–263.25) (*n* = 30)	0.022	0.977 (0.952–0.989)
Serum albumin (mg/L) (*n* = 30)	38,703.85 (35,763.43–42,831.53) (*n* = 30)	41,900 (39,975–45,325) (*n* = 30)	<0.001	0.828 (−0.34–0.956)

CSF and serum κFLC, CSF and serum albumin concentrations obtained with Freelite^®^-Optilite and N Latex^®^-BNII and results of the inter-method comparison in the whole cohort and in the different subgroups. κFLC, kappa free light chains; mg, milligram; L, liter; IQR, interquartile range; ICC, intraclass correlation coefficient; CI, confidence interval; MS, multiple sclerosis; OIND, other inflammatory or infectious neurological diseases of the central and peripheral nervous system; NIND, non-inflammatory neurological diseases; NR, not reported. The bold values represent the statistically significant findings.

There were no statistically significant differences in overall CSF κFLC levels between Freelite^®^-Optilite and N Latex^®^-BNII. However, serum κFLC, serum albumin, and CSF albumin levels significantly differed between the two methods (**Table 2**).

**Figure 2:**
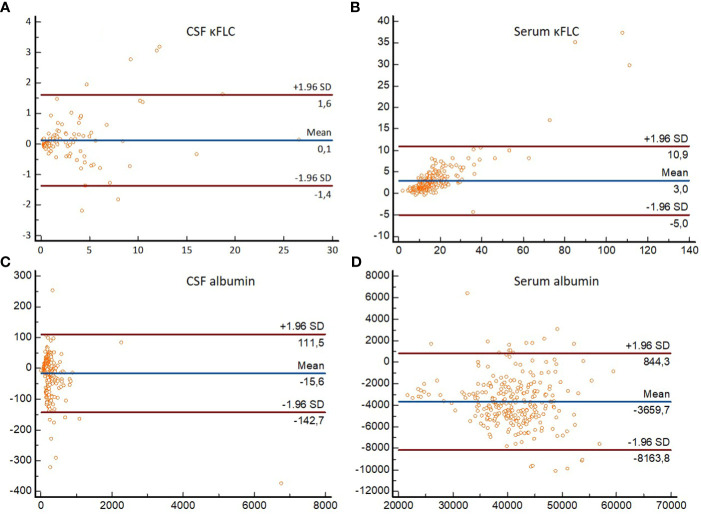
Bland–Altman plots demonstrating differences in absolute values obtained with Freelite^®^-Optilite and N Latex^®^-BNII for (A) CSF κFLC, (B) serum κFLC, (C) CSF albumin, and (D) serum albumin. For each participant, differences between the concentrations (mg/L) obtained with the two methods (*y*-axis) are plotted against the averages of the values (mg/L) obtained with the two methods (*x*-axis). Horizontal lines are drawn at the mean difference between the two methods, and at the limits of agreement, which are defined as the mean difference plus and minus 1.96 times the standard deviation of the differences. κFLC, kappa free light chains; SD, standard deviation.

Results of the Bland–Altman analysis for the whole cohort are displayed in **Figure 2**. Passing–Bablok regression analysis (**Table 3**) revealed no differences for CSF κFLC, both systematic (SyD) and proportional differences (PrD) for serum κFLC and SyD for serum albumin between the two methods. ICC demonstrated excellent agreement between Freelite^®^-Optilite and N Latex^®^-BNII for CSF κFLC and CSF albumin values, good agreement for serum albumin, and moderate agreement for serum κFLC concentrations (**Table 2**).

**Table 3:** Comparison between concentrations obtained with Freelite^®^-Optilite and N Latex^®^-BNII for serum and CSF κFLC and serum and CSF albumin using Passing–Bablok regression analysis.

	Passing and Bablok regression			
Whole cohort	Intercept A (95% CI)	Slope B (95% CI)	Residuals (95% CI)	Interpretation
CSF κFLC (mg/L)	−0.02729 (−0.07318 to 0.01)	1.0409 (1.000 to 1.0909)	0.5189 (to 1.017 to 1.017)	ND
Serum κFLC (mg/L)	−0.965 (−1.478 to −0.4875)	1.2283 (1.1925 to 1.2669)	20.693 (−40.5583 to 40.5583)	SyD, PrD
CSF albumin (mg/L)	NP	NP	NP	NP
Serum albumin (mg/L)	−2,160.9442 (−3,974.8157 to −513.2379)	0.9595 (0.9182 to 1.0041)	1,623.5418 (−3,182.142 to 3,182.142)	SyD
MS	Intercept A (95% CI)	Slope B (95% CI)	Residuals (95% CI)	Interpretation
CSF κFLC (mg/L)	−0.06475 (−0.1836 to 0.035)	1.062 (1 to 1.1373)	0.5817 (−1.1402 to 1.1402)	ND
Serum κFLC (mg/L)	−1.7144 (−2.7036 to −0.7818)	1.3257 (1.2333 to 1.4202)	0.9512 (−1.8644 to 1.8644)	SyD, PrD
CSF albumin (mg/L)	−5.334 (−46.8764 to 28.6306)	0.8489 (0.6582 to 1.0187)	43.3173 (−84.9018 to 84.9018)	ND
Serum albumin (mg/L)	−1,186.5766 (−6,201.3211 to 3,291.1118)	0.9363 (0.8346 to 1.0488)	1,883.6991 (−3,692.0502 to 3,692.0502)	ND
OIND	Intercept A (95% CI)	Slope B (95% CI)	Residuals (95% CI)	Interpretation
CSF κFLC (mg/L)	0.03399 (−0.06302 to 0.3143)	0.9871 (0.8286 to 1.1153)	0.6557 (−1.2852 to 1.2852)	ND
Serum κFLC (mg/L)	−1.0556 (−1.8555 to 0.01545)	1.2246 (1.1506 to 1.2911)	1.9818 (−3.8843 to 3.8843)	PrD
CSF albumin (mg/L)	16.3159 (2.4478 to 34.6193)	0.8931 (0.8296 to 0.9459)	68.4802 (−134.2212 to 134.2212)	SyD, PrD
Serum albumin (mg/L)	−387.2351 (−2,615.3041 to 1,260.6349)	0.9094 (0.8658 to 0.9704)	1,375.3497 (−2,695.6854 to 2,695.6854)	PrD
NIND	Intercept A (95% CI)	Slope B (95% CI)	Residuals (95% CI)	Interpretation
CSF κFLC (mg/L)	−0.02783 (−0.1147 to 0.03640)	1.0575 (0.9200 to 1.2118)	0.2225 (−0.4361 to 0.4361)	ND
Serum κFLC (mg/L)	−0.6871 (−1.4150 to 0.1317)	1.1976 (1.1424 to 1.2550)	34.5122 (−67.6439 to 67.6439)	PrD
CSF albumin (mg/L)	23.6538 (13.1706 to 36.5473)	0.9407 (0.8994 to 0.9930)	29.9974 (−58.7950 to 58.7950)	SyD, PrD
Serum albumin (mg/L)	−2,053.0883 (−5,442.4170 to 807.8872)	0.9579 (0.8906 to 1.0421)	1,604.6135 (−3,145.0425 to 3,145.042)	ND
Symptomatic controls	Intercept A (95% CI)	Slope B (95% CI)	Residuals (95% CI)	Interpretation
CSF κFLC (mg/L)	NP	NP	NP	NP
Serum κFLC (mg/L) (*n* = 30)	−1.4214 (−3.1130 to 0.3824)	1.2476 (1.1177 to 1.4034)	1.0195 (−1.9983 to 1.9983)	PrD
CSF albumin (mg/L) (*n* = 30)	3.1821 (−24.72 to 30.4351)	1.0679 (0.8864 to 1.2050)	35.2688 (−69.1269 to 69.1269)	ND
Serum albumin (mg/L) (*n* = 30)	−9,469.7193 (−18,072.8972 to −4,471.0833)	1.1399 (1.0187 to 1.3495)	1,379.8888 (−2,704.5820 to 2,704.5820)	SyD, PrD

Results of the comparison between concentrations obtained with Freelite^®^-Optilite and N Latex^®^-BNII for serum and CSF κFLC and serum and CSF albumin using Passing–Bablok regression analysis. In brief, the Passing–Bablok statistical procedure fits the parameters a and b of the linear equation y = a + b x using non-parametric methods where y represents the observations obtained by method 1 and x represents the observations obtained by method 2. This procedure is only valid when a linear relationship exists between x and y, which can be assessed by a cusum test. If no linear relationship existed between x and y, Passing–Bablok regression analysis was not performed, which is indicated as “NP”. The interpretation of the results is as follows: If 0 is in the CI of a, and 1 is in the CI b, the two methods are comparable within the investigated concentration range. If 0 is not in the CI of a, there is a systematic difference and if 1 is not in the CI of b, then there is a proportional difference between the two methods. The equation y = a + b x defines the regression line, but not all observations lie on that line. In fact, observations are rather defined by y = a + b x + e, where “e” represents the residual with the residual being the difference of the observed y value with the value predicted by the regression equation for the corresponding x value. Residuals therefore represent the remaining variation after correcting for systematic and proportional differences. κFLC, kappa free light chains; mg, milligram; L, liter; CI, confidence interval; MS, multiple sclerosis; OIND, other inflammatory or infectious neurological diseases of the central and peripheral nervous system; NIND, non-inflammatory neurological diseases; NP, not performed; ND, no difference; SyD, systematic difference; PrD, proportional difference.

Similarly, subgroup analysis revealed that serum κFLC, CSF albumin, and serum albumin concentrations significantly differed between the two methods in all subgroups, whereas no significant differences between Freelite^®^-Optilite and N Latex^®^-BNII for CSF κFLC measurements could be identified. Passing–Bablok regression analysis showed no differences for CSF κFLC between the two methods in all subgroups (**Table 3**). For serum κFLC, both SyD and PrD were shown in MS, whereas PrD were seen in OIND, NIND, and SC. SyD and PrD for CSF albumin levels were demonstrated in OIND and NIND, whereas no differences were observed in MS and SC. For serum albumin, no differences were demonstrated in MS and NIND, whereas PrD between the two methods were seen in OIND and both SyD and PrD in SC.

However, in all subgroups, excellent agreement between Freelite^®^-Optilite and N Latex^®^-BNII was shown for CSF κFLC and good reliability for serum albumin (**Table 2**). In contrast, reliability between the two methods ranged from poor (NIND) to good (MS and SC) and even excellent (OIND) for serum κFLC measurements, whereas for CSF albumin, it ranged from good (MS) to excellent (OIND, NIND, and SC) (**Table 2**).

### 3.3 Comparison of the κFLC index, κIgG index, and CSF κFLC/IgG ratio

Serum IgG concentrations [median, 10.10 g/L (IQR 8.23–11.90)] were available for 239 participants whereas CSF IgG [median, 3 mg/dl (IQR 2.1–4.6)] concentrations were available for 247 participants. Results of the method comparison for the κFLC index, κIgG index, and CSF κFLC/IgG ratio are presented in **Tables 4**, **5**. Similarly, reliability analysis for comparison between Freelite^®^-Optilite and N Latex^®^-BNII was performed twice: once with only measurable CSF κFLC concentrations (**Table 4**) and once where undetectable CSF κFLC concentrations were replaced by the detection limit (**Supplementary Table 3**).

**Table 4:** κFLC index, κIgG index, and CSF κFLC/IgG ratio: Comparison between Freelite^®^-Optilite and N Latex^®^-BNII.

Whole cohort	Freelite-Optilite (Bruges)	N Latex-BN II (Ghent)		
	Median (IQR)	Median (IQR)	*p*-value	ICC (95% CI)
κFLC index	18.96 (3.49–69.34) (*n* = 114)	20.86 (4.23–57.28) (*n* = 108)	0.757	0.939 (0.911–0.958)
κIgG index	33.60 (7.87–68.69) (*n* = 96)	36.19 (7.49–80.70) (*n* = 92)	**<0.001**	0.944 (0.892–0.968)
CSF κFLC/IgG ratio	5.39 (1.42–9.80) (*n* = 104)	4.94 (1.60–11.10) (*n* = 99)	0.435	0.954 (0.932–0.969)
MS	Freelite-Optilite (Bruges)	N Latex-BN II (Ghent)		
κFLC index	43.28 (18.35–102.36) (*n* = 73)	37.49 (17.80–87.15) (*n* = 68)	0.409	0.920 (0.874–0.95)
κIgG index	54.67 (29.16–90.27)(*n* = 62)	60.95 (35.01–102.66) (*n* = 59)	<0.001	0.917 (0.796–0.96)
CSF κFLC/IgG ratio	7.27 (3.75–14.45) (*n* = 68)	7.55 (3.50–13.38) (*n* = 64)	0.481	0.94 (0.904–0.963)
OIND	Freelite-Optilite (Bruges)	N Latex-BN II (Ghent)		
κFLC index	5.10 (1.85–18.91) (*n* = 16)	5.95 (1.95–24.69) (*n* = 15)	0.211	0.981 (0.945–0.993)
κIgG index	10.58 (3.59–32.46)(*n* = 14)	9.99 (4.94–30.68) (*n* = 13)	0.116	0.967 (0.896–0.99)
CSF κFLC/IgG ratio	5.24 (1.26–7.79) (*n* = 15)	3.90 (0.84–10.42) (*n* = 14)	0.807	0.92 (0.77–0.974)
NIND	Freelite-Optilite (Bruges)	N Latex-BN II (Ghent)		
κFLC index	1.52 (1.15–2.48) (*n* = 23)	1.75 (1.38–2.99) (*n* = 23)	0.005	0.985 (0.959–0.994)
κIgG index	3.51 (2.41–5.37)(*n* = 19)	3.25 (2.66–6.63) (*n* = 19)	0.018	0.955 (0.887–0.983)
CSF κFLC/IgG ratio	0.98 (0.62–1.45) (*n* = 19)	0.96 (0.56–1.56) (*n* = 19)	0.777	0.995 (0.987–0.998)
Symptomatic controls	Freelite-Optilite (Bruges)	N Latex-BN II (Ghent)		
κFLC index	NR (*n* = 2)	NR (*n* = 2)	NR (*n* = 2)	NR (*n* = 2)
κIgG index	NR (*n* = 1)	NR (*n* = 1)	NR (*n* = 2)	NR (*n* = 2)
CSF κFLC/IgG ratio	NR (*n* = 2)	NR (*n* = 2)	NR (*n* = 2)	NR (*n* = 2)

Results of the comparison between κFLC index, κIgG index, and CSF κFLC/IgG ratio values obtained with Freelite^®^-Optilite and N Latex^®^-BNII in the whole cohort and in the different subgroups. κFLC, kappa free light chains; IgG, immunoglobulin G; IQR, interquartile range; ICC, intraclass correlation coefficient; CI, confidence interval; MS, multiple sclerosis; OIND, other inflammatory or infectious neurological diseases of the central and peripheral nervous system; NIND, non-inflammatory neurological diseases; NR, not reported. The bold values represent the statistically significant findings.

**Table 5:** Comparison between values obtained with Freelite^®^-Optilite and N Latex^®^-BNII for the κFLC index, κIgG index, and CSF κFLC/IgG ratio using the Passing–Bablok regression analysis.

	Passing and Bablok regression			
Whole cohort	Intercept A (95% CI)	Slope B (95% CI)	Residuals (95% CI)	Interpretation
κFLC index	−0.04181 (−0.8695 to −0.1158)	1.0254 (0.9419 to 1.1164)	16.72 (−32.7712 to 32.7712)	SyD
κIgG index	−0.07213 (−0.7803 to 0.2584)	0.8577 (0.8144 to 0.9049)	9.3408 (−18.3079 to 18.3079)	PrD
CSF κFLC/IgG ratio	−0.03646 (−0.1447 to 0.04972)	1.0214 (0.9722 to 1.0778)	1.2858 (−2.5202 to 2.5202)	ND
MS	Intercept A (95% CI)	Slope B (95% CI)	Residuals (95% CI)	Interpretation
κFLC index	−2.3464 (−8.8843 to 1.0731)	1.1186 (0.9752 to 1.2838)	21.9865 (−43.0936 to 43.0936)	ND
κIgG index	−2.3478 (−6.0829 to 0.8079)	0.8901 (0.8141 to 0.9665)	11.4008 (−22.3455 to 22.3455)	PrD
CSF κFLC/IgG ratio	−0.2930 (−0.7698 to 0.08953)	1.0676 (0.9900 to 1.1687)	1.4118 (−2.7671 to 2.7671)	ND
OIND	Intercept A (95% CI)	Slope B (95% CI)	Residuals (95% CI)	Interpretation
κFLC index	0.1518 (−0.4617 to 0.6807)	0.8452 (0.7785 to 1.0123)	5.7275 (−11.2259 to 11.2259)	ND
κIgG index	−0.2698 (−3.5026 to 0.7403)	0.9122 (0.8238 to 1.4174)	6.4220 (−12.5871 to 12.5871)	ND
CSF κFLC/IgG ratio	0.3231 (−0.07698 to 0.8300)	0.8886 (0.7850 to 1.0962)	1.2409 (−2.4321 to 2.4321)	ND
NIND	Intercept A (95% CI)	Slope B (95% CI)	Residuals (95% CI)	Interpretation
κFLC index	−0.1712 (−0.6430 to 0.05273)	0.8842 (0.7727 to 1.1264)	0.3941 (−0.7724 to 0.7724)	ND
κIgG index	0.2408 (−0.6587 to 0.6068)	0.7741 (0.7156 to 0.9612)	0.7792 (−1.5272 to 1.5272)	PrD
CSF κFLC/IgG ratio	0 (−0.1155 to 0.09407)	1 (0.8704 to 1.1348)	0.1907 (−0.3738 to 0.3738)	ND
Symptomatic controls	Intercept A (95% CI)	Slope B (95% CI)	Residuals (95% CI)	Interpretation
κFLC index	NP	NP	NP	NP
κIgG index	NP	NP	NP	NP
CSF κFLC/IgG ratio	NP	NP	NP	NP

Results of the comparison between values obtained with Freelite^®^-Optilite and N Latex^®^-BNII for the κFLC index, κIgG index, and CSF κFLC/IgG ratios using Passing–Bablok regression analysis. In brief, the Passing–Bablok statistical procedure fits the parameters a and b of the linear equation y = a + b x using non-parametric methods where y represents the observations obtained by method 1 and x represents the observations obtained by method 2. This procedure is only valid when a linear relationship exists between x and y, which can be assessed by a cusum test. If no linear relationship existed between x and y, Passing–Bablok regression analysis was not performed, which is indicated as “NP”. The interpretation of the results is as follows: If 0 is in the CI of a, and 1 is in the CI b, the two methods are comparable within the investigated concentration range. If 0 is not in the CI of a, there is a systematic difference and if 1 is not in the CI of b, then there is a proportional difference between the two methods. The equation y = a + b x defines the regression line, but not all observations lie on that line. In fact, observations are rather defined by y = a + b x + e, where “e” represents the residual with the residual being the difference of the observed y value with the value predicted by the regression equation for the corresponding x value. Residuals therefore represent the remaining variation after correcting for systematic and proportional differences. κFLC, kappa free light chains; IgG, immunoglobulin G; CI, confidence interval; MS, multiple sclerosis; OIND, other inflammatory or infectious neurological diseases of the central and peripheral nervous system; NIND, non-inflammatory neurological diseases; NP, not performed; ND, no difference; SyD, systematic difference; PrD, proportional difference.

**Figure 3:**
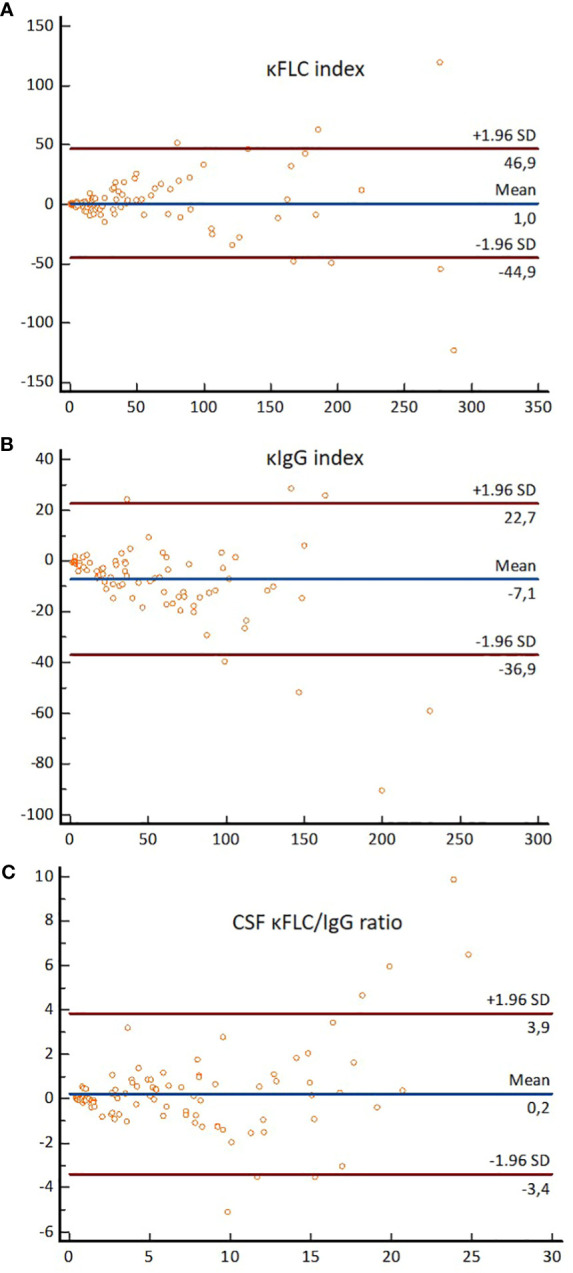
Bland–Altman plots demonstrating differences in absolute values obtained by Freelite^®^-Optilite and N Latex^®^-BNII for **(A)** the κFLC index, **(B)** the κIgG index, and **(C)** the CSF κFLC/IgG ratio. For each participant, differences between the values obtained with the two methods (*y*-axis) are plotted against the averages of the values obtained with the two methods (*x*-axis). Horizontal lines are drawn at the mean difference between the two methods and at the limits of agreement, which are defined as the mean difference plus and minus 1.96 times the standard deviation of the differences. κFLC, kappa free light chains; SD, standard deviation.

There were no statistically significant differences in κFLC index and CSF κFLC/IgG ratio values between Freelite^®^-Optilite and N Latex^®^-BNII in the whole cohort (**Table 4**). Similar results were seen in all subgroups except for NIND, where significant inter-method differences in κFLC index values were demonstrated. For the κIgG index, significant differences were seen in the whole cohort as well as in all subgroups except for OIND. Results of the Bland–Altman analysis for the whole cohort are displayed in **Figure 3**. Passing–Bablok regression analysis revealed PrD for the κIgG index in the whole cohort as well as the MS and NIND subgroups, whereas no differences were seen in OIND. SyD between the two methods were demonstrated for the κFLC index in the whole cohort, whereas no differences could be demonstrated in all subgroups (**Table 5**). For the CSF κFLC/IgG ratio, no differences between the two methods were observed in the whole cohort as well as in all subgroups. Reliability analysis showed excellent agreement between the two methods for the κFLC index, κIgG index, and CSF κFLC/IgG ratio in the whole cohort as well as in all subgroups, as demonstrated by an ICC >0.90 (**Table 4**).

### 3.4 Impact of age, sample storage duration, and corticosteroid administration on κFLC measures

We refer to **Supplementary Table 4** for the correlation results (Spearman rank correlation) between κFLC measures in relation to both age and storage duration. Since the κIgG index and CSF κFLC/IgG ratio were calculated using CSF and serum IgG levels determined at the time of sample collection, IgG levels could not be prone to storage duration. Therefore, correlation and multivariable analyses assessing the influence of sample storage duration on κFLC measures were not performed for the κIgG index and the CSF κFLC/IgG ratio. Similar to the method comparison analysis, only samples with measurable CSF κFLC levels were used for correlation and multivariable linear regression analyses.

Of note, in the entire cohort, a significant positive correlation was observed between age and serum κFLC levels for both Freelite^®^-Optilite and N Latex^®^-BNII. This correlation was consistent across all subgroups, except for SC. Conversely, a significant negative correlation was found between age and the κFLC index, κIgG index, and CSF κFLC/IgG ratio (Freelite^®^-Optilite and N Latex^®^-BNII). However, this association was not consistently seen within the different subgroups.

In the 14 days prior to LP, corticosteroids were administered in 21 participants, of whom 7 belonged to the MS group, 8 belonged to OIND, 5 belonged to NIND, and 1 belonged to SC. Multivariable linear regression analysis demonstrated that serum κFLC, CSF κFLC, and κFLC index values were not influenced by corticosteroid administration or sample storage duration (data not shown). On the other hand, serum κFLC levels measured with N Latex^®^-BNII increased with age [*B* = 0.176 (0.058–0.293), *p* = 0.004, adjusted *R*^2^ = 0.058]. However, this could not be demonstrated for Freelite^®^-Optilite. All multivariable linear regression analyses were adjusted for diagnosis (MS versus controls), age at the moment of LP, sample storage duration, sex, and administration of corticosteroids.

## 4 Discussion

CSF κFLC parameters have gained increasing interest as alternative markers reflecting intrathecal IgG synthesis, which is of particular relevance in the diagnostic workup of a person with suspected MS (7). However, various κFLC parameters have been proposed, and the optimal κFLC parameter to differentiate MS from controls remains a subject of ongoing debate. In addition, studies comparing cutoff values of various κFLC parameters obtained with different reagents and platforms were up until today scarce. Lack of consistent data on these topics currently hampers the use of the κFLC index and its alternative measures in routine clinical practice. This study was designed in an attempt to tackle these hurdles.

Our findings demonstrated excellent diagnostic accuracy of the κFLC index and κIgG index to differentiate MS from controls (consisting of OIND, NIND, and SC), irrespective of the method used for κFLC quantification. Diagnostic power for the CSF κFLC/IgG ratio and isolated CSF κFLC was good using Freelite^®^-Optilite and excellent using N Latex^®^-BNII. All κFLC measures further showed good diagnostic power to differentiate MS from OIND with Freelite^®^-Optilite, whereas the diagnostic power ranged from good (isolated CSF κFLC and CSF κFLC/IgG ratio) to excellent (κFLC index and κIgG index) for N Latex^®^-BNII. Our results agree with the findings of earlier studies (6–9, 33).

Interestingly, optimal cutoff values for isolated CSF κFLC to differentiate MS from controls were identical for Freelite^®^-Optilite and N Latex^®^-BNII. Given that isolated CSF κFLC demonstrated superior sensitivity yet inferior specificity compared to CSF OCBs, these may potentially represent a method-independent, more cost-efficient, initial screening method to identify PwMS, with comparable diagnostic performances between the two methods, as illustrated by the similar ROC curves, sensitivities, and specificities. These findings require validation in future multicenter studies. However, optimal cutoff values to distinguish MS from OIND differed between the two methods. In clinical practice, one of the primary reasons to perform CSF analysis is to rule out other neuro-inflammatory disorders, and our results suggest that isolated CSF κFLC is the least suitable parameter to serve this purpose, as demonstrated by the AUC.

Although the diagnostic performance ranged from good to excellent for isolated CSF κFLC, the ROC curves discriminating MS from controls were inferior compared to the κFLC index and κIgG index. The superior diagnostic performance of the κFLC index compared to isolated CSF κFLC was previously demonstrated (18–20). The diagnostic accuracies of the κFLC index and κIgG index were comparable within the two methods and exceeded that of the alternative κFLC parameters. However, since elevated κFLC concentrations in CSF could also be a consequence of barrier dysfunction and therefore diffusion from the serum, determination of serum κFLC and reference to albumin quotient seems necessary for accurate interpretation. In addition, given the overwhelming evidence supporting the high diagnostic accuracy of the κFLC index compared to the scarce data on the κIgG index, the κFLC index seems to be the measure of choice for the time being. However, as we confirmed the high diagnostic accuracy of the κIgG index (8), it seems essential to further explore this measure in future studies.

The detection limit for CSF κFLC was higher for Freelite^®^ than for N Latex^®^. In clinical practice, however, the κFLC index is reported as below the calculated numerical value in case of a CSF κFLC concentration below the detection limit, which poses no interpretational clinical problem as the optimal κFLC index cutoff value is 7.7 for Freelite^®^-Optilite.

Up until today, there is no consensus on which cutoff value to use for demonstrating MS-related intrathecal IgG synthesis. Published κFLC index cutoff values range from 2.4 to 20 (7) and depend on the chosen control population and on how undetectable CSF κFLC levels were dealt with. In our study, cutoff values that optimized sensitivity and specificity using the Youden index differed between Freelite^®^-Optilite and N Latex^®^-BNII. ROC curves (**Figure 1**) further show that the specificity of Freelite^®^-Optilite is slightly lower at sensitivities between 70% and 100% than N Latex^®^-BNII for all measures. Harmonization of clinical interpretation can, however, be obtained by calculating result-specific LR (34) (**Supplementary Table 1**).

First of all, these findings are of outmost importance for neurologists and clinical chemists that deal with PwMS in routine clinical practice. Furthermore, this observation is very pertinent when multicenter studies combine findings of different laboratories. For instance, in a recent large multicenter study including 1,621 patients, of whom 675 were diagnosed with MS, a κFLC index >8.92 could differentiate MS from alternative diagnosis whereas a κFLC index >11.56 could differentiate MS from other inflammatory neurological diseases (35). In this study, κFLC were quantified in 13 French MS centers using either the Optilite (n = 9) or the SPA Plus (n = 2) turbidometer (The Binding Site, Birmingham, UK) or the BNII (n = 1) or BN Prospec (n = 1) nephelometer (Siemens Healthcare Diagnostic Products). Their multivariable analysis showed that κFLC index values were not influenced by the κFLC analyzer type (35). However, this study was not designed to assess variation between methods, and their analysis might be biased by the fact that the majority of the centers used turbidimetry for κFLC quantification. In addition, since only Freelite^®^ was used for analysis, no statements could be made about inter-reagent variability in κFLC index values. Another multicenter study retrospectively collected κFLC index values of 174 patients with primary progressive MS, and could not demonstrate any impact of the type of platform or assay on κFLC index positivity, which was defined as a κFLC index value ≥6.1 (36). The median κFLC index value in this cohort was 54.6 (IQR 17.8–144.0). It is therefore not surprising that the applied method did not influence κFLC index positivity, as we demonstrated that optimal cutoff values were 7.70 and 4.71 with Freelite^®^-Optilite and N Latex^®^-BNII, respectively. Similarly, a recent meta-analysis could not identify any significant impact of assay and analytical platform on κFLC index values (33). However, these studies were not designed to assess between method variation in diagnostic accuracies and optimal cutoff values. Our findings clearly demonstrate that optimal diagnostic cutoff values differ between methods. Therefore, results on κFLC parameters cannot be interchanged when different reagents and analytical platforms are used. It is plausible that, in the future, the κFLC index will be incorporated in the diagnostic criteria for MS, and if this occurs, one must be aware that method-specific cutoff values should be used unless result-specific LR will be implemented. These results must also be kept in mind while conducting large-scale, multicenter studies using data from, for example, international MS registries.

Although the diagnostic power (AUC) of the κFLC index and the κIgG index exceeded that of CSF OCBs irrespective of the method used, the detection of CSF OCBs remained the most specific method to confirm a diagnosis of MS. These results confirm that the κFLC index and the κIgG index can serve as reliable alternative markers reflecting intrathecal IgG synthesis and further lend support to the future use of the “reflex approach”. In this proposed approach, two cutoff points should be applied to demonstrate intrathecal κFLC synthesis. Values below the lower cutoff should ensure that individuals with negative results indeed have no signs of intrathecal B-cell activity, whereas values exceeding the higher cutoff value should unequivocally identify those patients with intrathecal IgG synthesis. In case of values between the lower and higher cutoff, the so-called “gray zone”, CSF OCBs detection should follow intrathecal κFLC quantification (7). Thus, the lower cutoff value should approach a sensitivity of almost 100%, whereas for the higher cutoff value, specificity should be optimized.

Despite the use of different reagents and platforms, we demonstrated excellent agreement for the κFLC index, κIgG index, and CSF κ-FLC/IgG ratio between Freelite^®^-Optilite and N Latex^®^-BNII. In addition, excellent inter-method agreement was shown for CSF κFLC and CSF albumin concentrations. Serum albumin measurements showed less concordance compared to CSF albumin measurements (ICC of 0.80 and 0.99, respectively) and only moderate agreement between the two methods was shown for serum κFLC. Similar work was done by Natali et al. (37) and Zeman et al. (13), the former showing an excellent agreement of the κFLC index values among The Binding Site and Siemens laboratories, which is entirely consistent with our findings. However, the small sample size (*n* = 15) of their study required validation on a larger dataset such as ours. Natali et al. further demonstrated that the agreement was greater for CSF κFLC and albumin as opposed to serum measurements across all laboratories consisting of Binding Site (=Freelite^®^-SPAplus), Siemens (=N Latex^®^-BNProspec), and mixed laboratories (=Freelite^®^- BNProspec) (37). In line with our findings, Zeman et al. reported an excellent agreement for CSF κFLC levels (rho = 0.979) between Binding Site (Freelite^®^-SPAplus) and Siemens (N Latex^®^-BNprospec) laboratories, whereas—in contrast to our findings—good agreement for serum κFLC (rho = 0.865) measurements was demonstrated (13). Finally, good interassay CSF κFLC agreement (Freelite^®^-BN Prospec versus N Latex^®^-BN Prospec) (*r*^2^ = 0.86) was previously demonstrated by Susse et al. (38).

Although the agreement between N Latex^®^-BNII and Freelite^®^-Optilite ranged from moderate to excellent in our cohort, it is important to note that absolute values differed for all measures. This can probably be attributed to the use of different analytical methods, different reagents, and different platforms, resulting in different analytical sensitivity, measuring ranges, lower limits of quantitation, and reference ranges (13, 31). In patients with different plasma cell dyscrasias, for instance, the Freelite^®^ and N Latex^®^ reagents showed equivalent clinical performance (21–28); nevertheless, differences in absolute values, especially at high concentrations, have consistently been demonstrated (24, 25, 27–31). This was also shown in our study, where the agreement between N Latex^®^-BNII and Freelite^®^-Optilite was only moderate for serum κFLC measurements. Several reasons can be hypothesized for why this variability is observed. First, Freelite^®^ reagents consist of polyclonal antibodies, whereas N Latex^®^ consists of a mixture of monoclonal antibodies (39). Monoclonal FLCs show an abnormal degree of polymerization causing overestimation with Freelite^®^, as Freelite^®^ reacts better with dimeric FLC than N Latex^®^ (40, 41), and underestimation using N Latex^®^ due to binding site masking (39). This issue did not arise when assessing CSF κFLC agreement in our study, probably because κFLC concentrations in (non-inflammatory) CSF are generally low. This, in turn, raises another concern: CSF κFLCs were often so low that they could not be measured by Freelite^®^, hampering direct comparison between N Latex^®^-BNII and Freelite^®^-Optilite in the lower concentrations ranges. Of note, results reported as below the detection limit did not change the clinical interpretation.

Serum κFLC concentrations are known to be influenced by various patient-related factors (42–45). A recent study demonstrated that the administration of high-dose corticosteroids led to lower levels of serum κFLC levels, while CSF κFLC and κFLC index levels were unaffected (46). Although our multivariable analysis could not replicate these results, it needs to be noted that our study was not designed to investigate the impact of corticosteroid administration on κFLC measures. Unfortunately, we lacked crucial information on the exact dosage and duration for some participants who received corticosteroids prior to LP. Additionally, to properly investigate this, changes in κFLC levels before and after corticosteroid administration should ideally be explored, which could not be done in our study.

In line with earlier studies (45, 47, 48), we demonstrated that serum κFLC levels increased with age. While serum κFLC concentrations have shown to be stable in samples stored at −20°C for at least a year (49, 50), the stability of CSF κFLC remains unexplored. Our multivariable analysis demonstrated that sample storage duration (at −80°C) did not impact serum and CSF κFLC and κFLC index values. However, our study was not designed to assess serum and CSF κFLC stability. Future research could focus on this aspect by assessing potential changes in serum and CSF κFLC levels under various storage conditions and over different time durations. This could involve measuring same-sample κFLC levels at the time of sample collection and at subsequent time points after storage, using the same assay and analyzers to ensure consistency. Understanding fluctuations in κFLC levels over time and under various storage conditions could offer valuable insights into the optimal storage conditions for maintaining κFLC stability, which would enhance the reliability of κFLC measurements in both clinical and research settings.

This study has limitations. First, our different subgroups were not completely balanced. However, the control group consisting of OIND, NIND, and SC was sufficiently large to determine diagnostic cutoff values to differentiate MS from controls. Second, for the determination of diagnostic cutoff values, we assigned an empirical CSF κFLC value to those CSF samples with concentrations below the detection limit. However, only six patients with MS had undetectable CSF κFLC concentrations. Hence, determination of diagnostic MS cutoff values was probably not affected, in line with a recent systematic review and meta-analysis (33). Furthermore, as intrathecal κFLC synthesis might also be present in case of intrathecal IgA or IgM synthesis (33), it would have been more accurate to calculate the CSF κFLC/Ig ratio by the following formula: CSF κFLC/(CSF IgA + IgM + IgG). Unfortunately, CSF IgM and IgA concentrations were retrospectively collected from the electronic patient file, and were only determined in a small subset of participants during routine clinical care. Finally, we assessed the diagnostic accuracy of several κFLC parameters using two different methods in two different laboratories. To further explore the interlaboratory robustness and to exclude variations caused by different laboratory environments, the two methods should be applied in several different laboratories in future studies.

Future studies should further explore the associations between isolated CSF κFLC, the κFLC index, the κIgG index, and the CSF κFLC/Ig ratio with clinical variables at baseline and during follow-up. This could not be investigated in our study, as clinical variables were not systematically documented.

## 5 Conclusion

Our findings demonstrated the excellent diagnostic accuracy of the κFLC index and κIgG index to differentiate MS from controls, irrespective of the method used for κFLC quantification. These results suggest that the κFLC index and κIgG index may serve as reliable alternative markers reflecting intrathecal IgG synthesis. Furthermore, our analysis revealed that optimal diagnostic cutoff values to distinguish MS from controls differed between centers using different assays and analytical platforms for all measures except for isolated CSF κFLC. Isolated CSF κFLC may therefore potentially serve as a method-independent, more cost-efficient, initial screening measure to identify individuals with MS. However, these findings require validation in future multicenter studies.

## Data availability statement

Anonymized data not published within this article will be made available upon reasonable request from any qualified investigator directed to the corresponding author.

## Ethics statement

This study was approved by the “commissie medische ethiek” from Ghent university hospital and  the “commissie voor ethiek” from AZ Sint-Jan Brugge hospital. The studies were conducted in accordance with the local legislation and institutional requirements. The participants provided their written informed consent to participate in this study.

## Author contributions

CD: Conceptualization, Data curation, Formal analysis, Investigation, Methodology, Project administration, Visualization, Writing – original draft, Resources. PK: Conceptualization, Data curation, Formal analysis, Investigation, Methodology, Project administration, Resources, Supervision, Visualization, Writing – review & editing. MC: Conceptualization, Data curation, Methodology, Writing – review & editing. LV: Data curation, Writing – review & editing. LVH: Data curation, Writing – review & editing. MV: Conceptualization, Data curation, Formal analysis, Investigation, Methodology, Project administration, Resources, Supervision, Writing – review & editing. GL: Conceptualization, Data curation, Methodology, Resources, Supervision, Writing – review & editing, Investigation, Project administration.
