# Supplementary material for: Inter-assay diagnostic accuracy of cerebrospinal fluid kappa free light chains for the diagnosis of multiple sclerosis
Source: Front Immunol. 2024 Apr 30;15:1385231. doi: 10.3389/fimmu.2024.1385231 (PMC11091388; doi:10.3389/fimmu.2024.1385231)
Supplement: Supplementary file 1 [file Table_1.docx]

# Supplementary materials

Table 1

Title Cut-off values of the κFLC parameters corresponding with a LR closest to 5 using Freelite®-Optilite and N Latex®-BNII

Legend Diagnostic accuracy of the κFLC parameters obtained with Freelite®-Optilite and N Latex®-BNII. To facilitate inter-method comparison, the diagnostic cut-off values that correspond with the LR closest to 5 are displayed. Abbrevations: κFLC: kappa free light chains; mg: milligram; l: litre, IgG: Immunoglobulin G; MS: multiple sclerosis; OIND: other inflammatory or infectious neurological diseases of the central and peripheral nervous system; ROC: Receiver Operating Characteristics: CI: confidence interval; LR: likelihood ratio; AUC: Area under the curve; NP: not possible.

| **Freelite-Optilite (Bruges)** | | | | | | |
| --- | --- | --- | --- | --- | --- | --- |
| **MS vs no Ms** | | | | | | |
|  | **Cut-off from the ROC** | **Sensitivity** | **Specificity** | **LR** | **Youden index** | **AUC** |
| CSF κFLC (mg/l) | 0.41 | 84.8 | 82.9 | 4.96 | 0.677 | 0.897 (0.853-0.942) |
| κFLC index | 5.98 | 89.9 | 82.1 | 5.02 | 0.720 | 0.924 (0.884-0.965) |
| κIgG index | 11.30 | 92.5 | 81.7 | 5.05 | 0.742 | 0.929 (0.887-0.971) |
| CSF κFLC/IgG ratio | 1.97 | 81.3 | 83.5 | 4.93 | 0.649 | 0.882 (0.829-0.935) |
| **MS versus OIND** | | | | | | |
| CSF κFLC (mg/l) | NP | NP | NP | NP | NP | 0.819 (0.738-0.900) |
| κFLC index | 10.32 | 79.7 | 84 | 4.98 | 0.637 | 0.877 (0.813-0.941) |
| κIgG index | 19.27 | 77.6 | 83.7 | 4.76 | 0.613 | 0.888 (0.821-0.954) |
| CSF κFLC/IgG ratio | 5.77 | 54.7 | 88.9 | 4.93 | 0.436 | 0.827 (0.750-0.903) |
| **N Latex-BN II (Ghent)** | | | | | | |
| **MS vs no Ms** | | | | | | |
|  | **Cut-off from the ROC** | **Sensitivity** | **Specificity** | **LR** | **Youden index** | **AUC (95% CI)** |
| CSF κFLC (mg/l) | 0.36 | 90.5 | 81.8 | 4.97 | 0.723 | 0.912 (0.875-0.949) |
| κFLC index | 2.85 | 94.6 | 81.2 | 5.03 | 0.758 | 0.962 (0.940-0.984) |
| κIgG index | 5.42 | 95.3 | 80.7 | 4.94 | 0.76 | 0.961 (0.934-0.988) |
| CSF κFLC/IgG ratio | 1.02 | 92.9 | 81.2 | 4.94 | 0.74 | 0.935 (0.902-0.968) |
| **MS versus OIND** | | | | | | |
| CSF κFLC (mg/l) | NP | NP | NP | NP | NP | 0.823 (0.738-0.909) |
| κFLC index | 6.31 | 89.2 | 82 | 4.96 | 0.712 | 0.910 (0.852-0.968) |
| κIgG index | 11.10 | 92.2 | 81.4 | 4.96 | 0.736 | 0.915 (0.856-0.974) |
| CSF κFLC/IgG ratio | 2.64 | 81.4 | 82.2 | 4.57 | 0.637 | 0.86 (0.786-0.934) |

Table 2

Title: Overview of the number of MS participants with both positive CSF OCB and elevated κFLC parameters and discordant results according to optimal cut-off values as determined by maximization of the Youden index with Freelite®-Optilite and N Latex®-BNII

Legend: Overview of the number of MS participants with both positive CSF OCB and elevated κFLC parameters and discordant results according to optimal cut-off values as determined by maximization of the Youden index with Freelite®-Optilite and N Latex®-BNII. Abbrevations: OCB: oligoclonal bands; +: positive; -: negative; κFLC: kappa free light chains; IgG: Immunoglobulin G; CSF: cerebrospinal fluid

|  | **Freelite-Optilite (Bruges)** | **N Latex-BN II (Ghent)** |
| --- | --- | --- |
| OCB+ and elevated CSF κFLC, n | 64 | 60 |
| OCB- and elevated CSF κFLC, n | 7 | 7 |
| OCB+, no elevated CSF κFLC, n | 2 | 1 |
| OCB-, no elevated CSF κFLC, n | 6 | 6 |
| OCB+ and elevated κFLC index, n | 65 | 61 |
| OCB- and elevated κFLC index, n | 6 | 9 |
| OCB+, no elevated κFLC index, n | 1 | 0 |
| OCB-, no elevated κFLC index, n | 7 | 4 |
| OCB+ and elevated κIgG index, n | 56 | 52 |
| OCB- and elevated κIgG index, n | 5 | 7 |
| OCB+, no elevated κIgG index, n | 1 | 2 |
| OCB-, no elevated κIgG index, n | 5 | 3 |
| OCB+ and elevated CSF κFLC/IgG ratio, n | 58 | 56 |
| OCB- and elevated CSF κFLC/IgG ratio, n | 3 | 8 |
| OCB+, no elevated CSF κFLC/IgG ratio, n | 5 | 2 |
| OCB-, no elevated CSF κFLC/IgG ratio, n | 9 | 4 |

Table 3:

Title: CSF κFLC, κFLC index, κIgG index and CSF κFLC/IgG ratio: Comparison between Freelite®-Optilite and N Latex®-BNII

Legend: CSF κFLC concentrations, κFLC index, κIgG index and CSF κFLC/IgG ratios obtained with Freelite®-Optilite and N Latex®-BNII and results of the inter-method comparison in the whole cohort and in the different subgroups. Undetectable CSF κFLC concentrations were replaced by the detection limit. Abbreviations: κFLC: kappa free light chains; mg: milligram; l: litre ,IQR: interquartile rang; ICC: intraclass correlation coefficient; CI: confidence interval; MS: multiple sclerosis; OIND: other inflammatory or infectious neurological diseases of the central and peripheral nervous system; NIND: non-inflammatory neurological diseases.

| **Whole cohort** | **Freelite-Optilite (Bruges)** | **N Latex-BN II (Ghent)** |  | |
| --- | --- | --- | --- | --- |
|  | **Median (IQR)** | **Median (IQR)** | **P value** | **ICC (95% CI)** |
| CSF κFLC (mg/l) | 0.27 (0.27-1.33) (n=260) | 0.25 (0.12-1.17) (n=255) | **<0.001** | 0.986 (0.981-0.989) |
| κFLC index | 3.91 (2.09-15.16) (n=258) | 2.22 (1.62-15.06) (n=255) | **<0.001** | 0.948 (0.934-0.959) |
| κIgG index | 8.28 (4.53-23.21) (n=231) | 4.24 (2.89-25.11) (n=230) | 0.056 | 0.955 (0.942-0.965) |
| CSF κFLC/IgG ratio | 1.35 (0.89-4.19) (n=245) | 0.69 (0.46-3.45) (n=240) | **<0.001** | 0.961 (0.941-0.973) |
| **MS** | **Freelite-Optilite (Bruges)** | **N Latex-BN II (Ghent)** |  | |
| CSF κFLC (mg/l) | 2.35 (0.85-4.59) (n=79) | 1.92 (0.88-4.72) (n=74) | 0.291 | 0.982 (0.971-0.989) |
| κFLC index | 39.61 (14.36-95.27) (n=79) | 33.15 (15.13-79.92) (n=74) | 0.452 | 0.925 (0.884-0.952) |
| κIgG index | 51.29 (23.12-79.35) (n=67) | 56.91 (29.85-98.33) (n=64) | **<0.001** | 0.925 (0.830-0.962) |
| CSF κFLC/IgG ratio | 6.61 (2.86-13.26) (n=75) | 6.49 (3.03-12.72) (n=70) | 0.394 | 0.947 (0.917-0.967) |
| **OIND** | **Freelite-Optilite (Bruges)** | **N Latex-BN II (Ghent)** |  | |
| CSF κFLC (mg/l) | 0.27 (0.27-0.56) (n=50) | 0.20 (0.1-0.53) (n=50) | **<0.001** | 0.987 (0.977-0.992) |
| κFLC index | 3.31 (1.90-6.99) (n=50) | 2.05 (1.47-4.44) (n=50) | **0.012** | 0.947 (0.908-0.970) |
| κIgG index | 6.43 (3.69-12.30) (n=43) | 4.14 (3.09-7.57) (n=43) | **0.039** | 0.943 (0.893-0.969) |
| CSF κFLC/IgG ratio | 1.00 (0.75-2.64) (n=45) | 0.59 (0.41-1.90) (n=45) | **<0.001** | 0.933 (0.880-0.963) |
| **NIND** | **Freelite-Optilite (Bruges)** | **N Latex-BN II (Ghent)** |  | |
| CSF κFLC (mg/l) | 0.27 (0.27-0.27) (n=101) | 0.17 (0.01-0.28) (n=101) | **<0.001** | 0.888 (0.684-0.948) |
| κFLC index | 2.74 (1.54-3.92) (n=99) | 1.77 (1.48-2.27) (n=101) | **<0.001** | 0.798 (0.660-0.875) |
| κIgG index | 5.68 (3.74-8.39) (n=94) | 3.17 (2.60-4.63) (n=96) | **<0.001** | 0.723 (0.502-0.837) |
| CSF κFLC/IgG ratio | 1.11 (0.78-1.47) (n=97) | 0.55 (0.43-0.85) (n=97) | **<0.001** | 0.837 (0.353-0.937) |
| **Symptomatic controls** | **Freelite-Optilite (Bruges)** | **N Latex-BN II (Ghent)** |  | |
| CSF κFLC (mg/l) | 0.27 (0.27-0.27) (n=30) | 0.11 (0.06-0.16) (n=30) | **<0.001** | 0.943 (0.106-0.987) |
| κFLC index | 3.45 (2.28-5.82) (n=30) | 1.83 (1.41-2.19) (n=30) | **<0.001** | 0.100 (-0.122-0.363) |
| κIgG index | 6.61 (4.93-9.29) (n=27) | 3.08 (2.25-3.71) (n=27) | **<0.001** | 0.123 (-0.107-0.399) |
| CSF κFLC/IgG ratio | 1.09 (0.90-1.48) (n=28) | 0.47 (0.34-0.63) (n=28) | **<0.001** | 0.055 (-0.125-0.297) |

Table 4:

Title: Correlation analyses between various κFLC measures in relation to both age and sample storage duration

Legend: Correlation between various κFLC measures in relation to both age and sample storage duration for the 2 methods. Results are displayed for the whole cohort and in the different subgroups. Only detectable CSF κFLC concentrations were used for analysis. Abbreviations: r= spearman rank correlation coefficient; κFLC: kappa free light chains; mg: milligram; l: litre; MS: multiple sclerosis; OIND: other inflammatory or infectious neurological diseases of the central and peripheral nervous system; NIND: non-inflammatory neurological diseases; NP: not performed.

| **Whole cohort** | **Freelite-Optilite (Bruges)** | | **N Latex-BN II (Ghent)** | |
| --- | --- | --- | --- | --- |
|  | **Age** | **Storage duration** | **Age** | **Storage duration** |
| Serum κFLC (mg/l) | **r=0.384, p<0.001**  (n=261) | **r= -0.149, p=0.016** (n=261) | **r=0.412, p<0.001**  (n=263) | **r= -0.164, p=0.008** (n=263) |
| CSF κFLC (mg/l) | r= -0.173, p=0.066 (n=114) | **r=0.189, p=0.044** (n=114) | r=-0.084, p=0.183 (n=251) | **r=0.301, p<0.001** (n=251) |
| κFLC index | **r= -0.416, p<0.001** (n=114) | **r=0.364, p<0.001** (n=114) | **r=-0.349, p<0.001** (n=251) | **r=0.366, p<0.001** (n=251) |
| κIgG index | **r= -0.457, p<0.001** (n=96) | NP | **r=-0.360, p<0.001** (n=227) | NP |
| CSF κFLC/IgG ratio | **r= -0.290, p=0.003** (n=104) | NP | **r=-0.167, p=0.010** (n=237) | NP |
| **MS** | **Freelite-Optilite (Bruges)** | | **N Latex-BN II (Ghent)** | |
|  | **Age** | **Storage duration** | **Age** | **Storage duration** |
| Serum κFLC (mg/l) | **r=0.418, p<0.001**  (n=80) | r= -0.102, p=0.370 (n=80) | **r=0.369, p<0.001**  (n=80) | r= -0.090, p=0.425 (n=80) |
| CSF κFLC (mg/l) | r=0.222, p=0.059 (n=73) | r= -0.062, p=0.605 (n=73) | r=0.026, p=0.823 (n=74) | r=-0.099, p=0.402 (n=74) |
| κFLC index | r=0.092, p=0.441 (n=73) | r= -0.088, p=0.458 (n=73) | r=-0.104, p=0.379 (n=74) | r=-0.079, p=0.505 (n=74) |
| κIgG index | r=0.116, p=0.369 (n=62) | NP | r=-0.014, p=0.910 (n=64) | NP |
| CSF κFLC/IgG ratio | r= 0.127, p= 0.303 (n=68) | NP | r=-0.066, p=0.589 (n=70) | NP |
| **OIND** | **Freelite-Optilite (Bruges)** | | **N Latex-BN II (Ghent)** | |
|  | **Age** | **Storage duration** | **Age** | **Storage duration** |
| Serum κFLC (mg/l) | **r=0.369, p=0.008** (n=51) | r= -0.131, p=0.359 (n=51) | **r=0.410, p=0.003** (n=51) | r= -0.105, p=0.464 (n=51) |
| CSF κFLC (mg/l) | r=0.004, p=0.987 (n=16) | r=0.349, p=0.185 (n=16) | **r=0.286, p=0.049** (n=48) | r=0.122, p=0.409 (n=48) |
| κFLC index | r=0.066, p=0.807 (n=16) | r=0.175, p=0.517 (n=16) | r=0.165, p=0.262 (n=48) | r=0.072, p=0.625 (n=48) |
| κIgG index | **r= -0.713, p=0.004 (n=14)** | NP | r=-0.114, p=0.472 (n=42) | NP |
| CSF κFLC/IgG ratio | r=0.120, p=0.671 (n=15) | NP | r=0.231, p=0.132 (n=44) | NP |
| **NIND** | **Freelite-Optilite (Bruges)** | | **N Latex-BN II (Ghent)** | |
|  | **Age** | **Storage duration** | **Age** | **Storage duration** |
| Serum κFLC (mg/l) | **r=0.354, p<0.001**  (n=100) | r= -0.063, p=0.533 (n=100) | **r=0.366, p<0.001** (n=102) | r= -0.074, p=0.460 (n=102) |
| CSF κFLC (mg/l) | r= -0.136, p=0.536 (n=23) | r= -0.271, p=0.211 (n=23) | r=0.359, p<0.001 (n=100) | r=-0.093, p=0.355 (n=100) |
| κFLC index | r= -0.204, p=0.352 (n=23) | r= -0.334, p=0.119 (n=23) | r=-0.206, p=0.040 (n=100) | r=-0.040, p=0.696 (n=100) |
| κIgG index | r= -0.086, p=0.726 (n=19) | NP | r=-0.109, p=0.295 (n=95) | NP |
| CSF κFLC/IgG ratio | r= -0.193, p=0.429 (n=19) | NP | r=0.271, p=0.008 (n=96) | NP |
| **Symptomatic controls** | **Freelite-Optilite (Bruges)** | | **N Latex-BN II (Ghent)** | |
|  | **Age** | **Storage duration** | **Age** | **Storage duration** |
| Serum κFLC (mg/l) | r= -0.155, p=0.413 (n=30) | r=0.208, p=0.270 (n=30) | r= -0.156, p=0.409 (n=30) | r=0.140, p=0.46 (n=30) |
| CSF κFLC (mg/l) | NP | NP | **r=0.406, p=0.029** (n=29) | r=-0.058, p=0.764 (n29) |
| κFLC index | NP | NP | r=0.174, p=0.367 (n=29) | r=-0.185, p=0.337 (n=29) |
| κIgG index | NP | NP | r=0.236, p=0.245 (n=26) | NP |
| CSF κFLC/IgG ratio | NP | NP | r=0.373, p=0.055 (n=27) | NP |
